# Effects of Wheat Oligopeptide on the Baking and Retrogradation Properties of Bread Rolls: Evaluation of Crumb Hardness, Moisture Content, and Starch Crystallization

**DOI:** 10.3390/foods13030397

**Published:** 2024-01-26

**Authors:** Yuting Zhang, Xiaorong Liu, Junbo Yu, Yang Fu, Xiangjun Liu, Ku Li, Dongfang Yan, Francisco J. Barba, Emlia Ferrer, Xuedong Wang, Jianjun Zhou

**Affiliations:** 1 Key Laboratory for Deep Processing of Major Grain and Oil, Ministry of Education, Hubei Key Laboratory for Processing and Transformation of Agricultural Products, Wuhan Polytechnic University, Wuhan 430023, China; yuuting2017@163.com (Y.Z.); 18253562371@163.com (X.L.); 15972083170@163.com (Y.F.); 2 Chinese Cereals and Oils Association, Beijing 100032, China; zglyxyjb@163.com; 3 National Key Laboratory of Agricultural Microbiology, Wuhan 430070, China; liuxjc@angelyeast.com (X.L.); liku@angelyeast.com (K.L.); yandf@angelyeast.com (D.Y.); 4 Research Group in Innovative Technologies for Sustainable Food (ALISOST), Nutrition, Food Science and Toxicology Department, Faculty of Pharmacy, Universitat de València, Avda. Vicent Andrés Estellés, s/n, Burjassot, 46100 València, Spain; francisco.barba@uv.es (F.J.B.); emilia.ferrer@uv.es (E.F.)

**Keywords:** wheat oligopeptide, bakery goods, starch crystallization, retrogradation properties, microstructure, storage process

## Abstract

Delaying the deterioration of bakery goods is necessary in the food industry. The objective of this study was to determine the effects of wheat oligopeptide (WOP) on the qualities of bread rolls. The effects of WOP on the baking properties, moisture content, and starch crystallization of rolls during the storage process were investigated in this study. The results showed that WOP effectively improved the degree of gluten cross-linking, thereby improving the specific volume and the internal structure of rolls. The FTIR and XRD results showed that the addition of WOP hindered the formation of the starch double helix structure and decreased its relative crystallinity. The DSC results revealed a decrease in the enthalpy change (ΔH) from 0.812 to 0.608 J/g after 7 days of storage with 1.0% WOP addition, further indicating that WOP reduced the availability of water for crystal lattice formation and hindered the rearrangement of starch molecules. The addition of WOP also improved the microstructure of the rolls that were observed using SEM analysis. In summary, WOP is expected to be an effective natural additive to inhibit starch staling and provide new insights into starchy food products.

## 1. Introduction

Bread rolls are one of the most important foods for humans, and their quality and sales are highly relevant as it will influence people’s consumption choices. The quality deterioration of bread rolls during the storage process causes severe economic losses and product waste worldwide [1]. For example, bread roll staling is manifested as increased tissue hardness and reduced product acceptability, which is caused by a series of physical and chemical changes during the storage process [2]. 

Until now, bread roll staling has remained a hot topic of research, despite advances in the staling mechanism of bread in recent years. The most important theories responsible for this phenomenon are moisture change and starch retrogradation in roll crumbs [3]. On the one hand, the drying of bread rolls during the storage process was mainly associated with moisture change from crumb to crust. More specifically, water is partially integrated into amylopectin during the storage process, which causes bread roll staling [4]. On the other hand, the gelatinized starch after baking at high temperature forms hydrogen bonds during storage, and the starch chains rearrange to form an ordered structure during the storage process [5]. Meanwhile, amylopectin molecules in the expanded starch granules bind to each other, resulting in increased rolls hardness and staling. The uniformity and stability of the microstructure of rolls will also be affected by these interactions, thus affecting the quality of the rolls [6].

Based on the previous research, the current strategy is to find appropriate ways to suppress the bread roll staling. At present, the feasible solution is to add different kinds of exogenous substances to delay the staling of rolls [7,8]. In recent years, protein and its hydrolysates have been proposed for their excellent physical and chemical properties and their potential in processing. For example, one study showed that *Listeria grass* carp protein hydrolysate could inhibit starch retrogradation, which was attributed to the fact that *Listeria grass* carp protein hydrolysate reduced the formation of hydrogen bonds of gelatinized starch during the storage process [9]. This indicates the effective role of protein hydrolysates in delaying the staling of starch products. Moreover, other reports also showed that the addition of lupine protein isolate enhanced the resistance to deformation and the extensibility of the dough and delayed bread roll staling [10]. Similarly, another study indicated that γ-polyglutamic acid could be used as a hydrophilic agent to improve the network structure of gluten, reduce water mobility, and increase the water retention of gluten [11].

Wheat oligopeptide (WOP) is a peptide mixture obtained by the enzymatic hydrolysis of wheat protein. Compared with wheat protein, the functional characteristics and intestinal absorption of wheat protein hydrolysates have been greatly improved. Moreover, WOP, as a new resource food, contains abundant nutrients and bioactive compounds with low molecular sizes [12]. Previous research has shown, through in vivo experiments, that WOP has the potential to alleviate aging-mediated intestinal mucosal oxidative stress and inflammation by fortifying the intestinal mucosal barrier [13]. Furthermore, it may play a role in enhancing the body’s natural defense mechanisms and supporting immune function. These biological functions suggest the potential applications of WOP in functional foods and dietary supplements [14]. In our previous studies, we carried out research focused on the interaction between WOP and wheat starch (WS) and indicated that WOP could effectively inhibit the staling behavior of WS [15]. However, there have been no reports investigating the effect of WOP on the quality of baked products until now. We speculate that WOP may improve baking quality and regulate staling by affecting starch properties and dough formation; therefore, the mechanism by which WOP affects the quality of bread rolls is worthy of further study.

This study determined the effects of WOP on WS bakery products, including the qualities of bread rolls, water change, and starch retrogradation during the storage process. The impacts of WOP on WS retrogradation in terms of thermal stability and long-range and short-range molecular order degree were investigated. In addition, the staling kinetics model was established to explore the effect of WOP on the bread roll staling properties. Overall, this study will provide a reference for the future application of WOP in the baking industry.

## 2. Materials and Methods

### 2.1. Materials

Golden statue wheat flour (WF) was purchased from Shekou Lam Soon Flour Mills Co., Ltd. (Shenzhen, China), with 12.10%, 0.36%, and 15.63% of moisture, ash, and crude protein, respectively. The wheat oligopeptide (WOP) (90% purity, food grade) was purchased from Xi’an BaiChuan Biological Technology (Xi’an, China). The high-activity dry yeast was provided by Angel Yeast (Yichang, China). The sugar, salt, and butter were obtained from the local market.

### 2.2. Breadmaking Process

Basic bread roll recipe consisted of 88% WF, 5.0% sugar, 5.0% butter, 1.0% yeast, and 1.0% salt. The WOP was used to partially replace WF in the formulation at varying amounts (0, 0.5%, 1.0%, and 1.5%) and integrated into the mixed flour. The mixed flour, sugar, yeast, and water (about 64.0%, based on dry basis) were mixed in a spiral mixer for 3 min to form the dough. The mixture was stirred, and then, the salt and butter were added until the gluten developed to make a smooth and elastic dough. The dough was placed in a fermentation room at 30 °C and 80% humidity for 30 min. The dough was then divided into 80 g pieces and leavened again for 60 min before baking at 200 °C for 15 min. It was then cooled for 1 h at room temperature (25 °C) for specific volume and hardness analysis. In addition, the rolls cooled for 1 h at room temperature (25 °C) above were packed in a polypropylene bag and stored at 4 °C for the analysis of bread staling. Six rolls were prepared in parallel for each group, and three replicate experiments were performed.

### 2.3. Evaluation of the Qualities of Bread Rolls: Specific Volume, Slice Structure, and Hardness

#### 2.3.1. Specific Volume

After rolls were left for 1 h at room temperature, the specific volume of rolls was measured (at 25 °C) by a food volume meter (BVW-L370, Perten, Sweden) according to the previous experimental methods [16].

The specific volume was calculated through Equation (1):

(1) $$Specific\;volume\;\left({cm}^{3}/g\right)=Bread\;rolls\;volume\left({cm}^{3}/g\right)/Bread\;rolls\;weight\left(g\right)$$

#### 2.3.2. Slice Structure Tests (C-Cell)

The slice structure test was performed using a baked product C-Cell imaging system (Sweden Botong Instruments, Stockholm, Sweden). According to a related study, the bread rolls were sliced into 15 mm thick slices from the center and placed in the collection box of a food image analyzer for photography [17]. Each sample was tested 3 times.

#### 2.3.3. Hardness Analysis and Staling Kinetics

The hardness of roll crumbs during storage from 0 to 7 days was measured with a texture analyzer (Bosin, Shanghai, China) equipped with a P/36R probe. Each roll crumb that was cut from center and was about 20 mm in thickness. The pre-test, test, and post-test rates were 3.0 mm/s, 1.0 mm/s, and 2.0 mm/s, respectively. The trigger force was 5 g, the shape variable was 50%, and the interval time was 2 s [18]. Each sample was repeated 3 times. The hardness under different storage periods were fitted to the ‘Avrami model’ to calculate the staling rates of samples following the research method [6], and the Avrami equation was as follows:

*R* = (*F_t_* − *F*_0_)/(*F*_∞_ − *F*_0_) = 1 − *exp*^^−*ktn*^ (2)

where ‘*R*’ is the crystallinity; ‘F_t_’, ‘F_0_’, and ‘F_∞_’ represent the retrogradation values of retrogradation at specific time (t), initial time (0), and limited time (∞), respectively; ‘k’ is the rate constant of recrystallization; and ‘n’ is the Avrami exponent.

Equation (3) can be transformed by converting Equation (2) as follows:

*In*[−*In*(1 – *R*)] = *nInt* + *Ink* (3)

‘n’ (Avrami exponent) and ‘k’ (bread roll staling rate constant) should be calculated according to formula (3).

### 2.4. Moisture Content Determination

The moisture content of roll crumb was determined according to the AACC Method of No:44-15A (2000). The thin slices of rolls were obtained from the center of the bread rolls, weighed accurately, and dried in the oven at 105 °C until a constant weight was obtained. The moisture content is expressed as the ratio of the weight of lost moisture to the initial weight of the sample:

(4) $$Moisture\;content=\left[Initial\;weight\;\left(g\right)-Final\;weight\left(g\right)\right]/Initial\;weight\;\left(g\right)$$

### 2.5. Retrogradation Properties Analysis

#### 2.5.1. Differential Scanning Calorimetry Analysis

The thermal properties of the samples were determined using a differential scanning calorimeter (DSC, Q2000, New Castle, DE, USA). The method was adjusted appropriately based on a previous study [19]. The enthalpy change (ΔH) was recorded with scanning the sample from 25 °C to 90 °C at a rate of 10 °C/min under nitrogen protection (20 mL/min).

#### 2.5.2. Long-Range Ordered Structure Analysis Using XRD Method

The X-ray diffractometer (XRD) method was modified according to a previous study [20]. The rolls were freeze-dried, and then, the samples were milled to pass through a 100-mesh sieve and analyzed using an XRD analyzer (Empyrean, PANalytical, Almelo, The Netherlands). The diffraction angle was scanned from 4° to 40°, and the speed was 4°/min under the conditions of 40 kV and 40 mA. The peaks and relative crystallinity of the images were analyzed with the Jade 6.5 software.

#### 2.5.3. Short-Range Ordered Structure Analysis Using FTIR

The freeze-dried bread roll powder was mixed with dry spectroscopic-grade potassium bromide powder in a mass ratio of 1:100. The ground sample was pressed into tablets and placed on the sample rack of FTIR (Thermo Nicolet NEXUS670, Thermo Fisher Science Inc., Waltham, MA, USA). The sample was scanned over the range of 4000 to 400 cm^−1^ with 32 scans and 4 cm^−1^ resolution [21].

#### 2.5.4. Scanning Electron Microscopy (SEM) Analysis

The bread rolls were sliced into cubes with sharp blades and freeze-dried. The cross-section of samples was sprayed with gold and fixed on the stage. The microstructure of the sample was observed with a scanning electron microscope (SEM, S-3000 N, Hitachi, Japan) at an accelerating voltage of 3 kV with the magnification of 2000× [16].

### 2.6. Statistical Analysis

The SPSS software (version 21.0, SPSS Inc., Chicago, IL, USA) was used for statistical analysis. Duncan’s multiple range test (*p* < 0.05) was used to evaluate the significant difference between the mean values. The graphs were drawn using origin 8.0 (version 8.0, Stat-Ease Inc., Minneapolis, MN, USA).

## 3. Results

### 3.1. Effects of WOP on the Qualities of Bread Rolls

#### 3.1.1. Specific Volume of Bread Rolls

**Figure 1:**
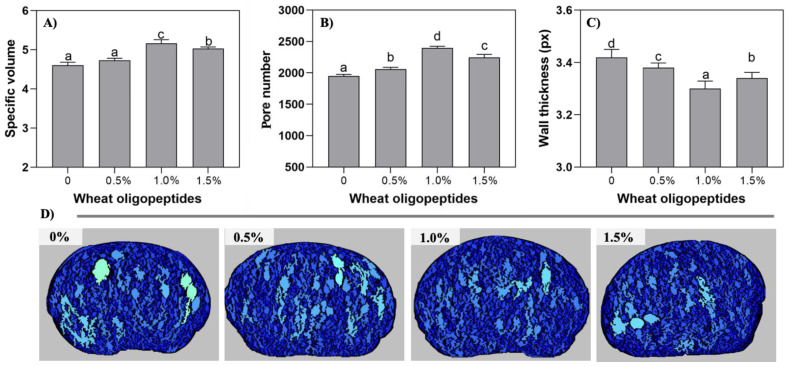
The visual status of the bread rolls with the addition of WOP (0–1.5%, *w*/*w*): (**A**) specific volume, (**B**) number of pores, (**C**) thickness of pore walls, and (**D**) bread roll cross-section C-Cell image. Different lowercase means significant difference between different groups (*p* < 0.05).

The specific volume of bread rolls is the most intuitive index to evaluate the baking quality of bread rolls, which is affected by the development of dough, and is also an important index to evaluate the synergistic effect of gluten network and yeast production capacity [5]. The effect of the addition of WOP (0, 0.5%, 1.0%, and 1.5%) on the specific volume of bread rolls is shown in Figure 1A.

From the results, it is evident that the addition of WOP increased the specific volume of bread rolls, depending on the amount of WOP added. Specifically, adding 0–0.5% of WOP had no significant effect on the specific volume of bread rolls (*p* > 0.05), but when the addition reached 1.0%, it significantly (*p* < 0.05) increased the specific volume of bread rolls. Overall, the best addition amount was 1.0%. Our data showed that WOP had a positive effect on the specific volume of bread rolls, which could be related to the ability of the gluten network to hold CO_2_ gas. The gluten network consisted of starch, gluten, and other ingredients. The CO_2_ generated during dough fermentation process, thereby increasing the volume of the bread rolls [22]. According to the related studies, these results could be attributed to the formation of hydrophilic complexes between WOP and gluten and starch, which increased the degree of cross-linking of gluten, promoting the better formation of gluten network structure and improving the air holding capacity and expansion capacity of the dough [23]. This was also consistent with a recent study, showing that whey protein increased the viscosity of the dough and the strength of the expanded cells, thereby improving the gas retention rate during baking and resulting in a better specific volume [24]. In addition, it has been suggested that yeast strains have the ability to effectively utilize non-sugar-based substrates, assimilating and degrading amino acids in the dough to obtain carbon and nitrogen units [25]. Therefore, WOP may be used as a nitrogen source for yeast, which improves their gas production capacity. Overall, combined with our research, the addition of WOP could increase the specific volume of the bread rolls through different mechanisms, ultimately improving the quality of bread rolls.

#### 3.1.2. Image C-Cell Analysis of Bread Roll Crumbs

The specific volume value of bread rolls directly reflects their volume; however, there are limitations as it cannot disclose the specific number of pores and the uniformity of the bread rolls’ internal structure. The C-Cell analysis can determine the quality of fermented products more clearly by quantifying the number and size of pores in bread roll slices [6].

The results in Figure 1B,D showed the internal structure of bread rolls with or without the WOP addition. Crumb structure analysis illustrated that the presence of WOP resulted in bread rolls with a small and uniform pore structure and a thinner pore wall thickness. Within the range of 0–1.0% WOP addition, the number of pores in the bread rolls increased with the WOP concentration (*p* < 0.05), while the thickness of the cell wall decreased significantly (*p* < 0.05). Generally, the increase in the pores number and the decrease in the pore wall thickness are positively correlated with the fineness of the bread rolls, which also means that WOP could facilitate to form softer, delicate, and elastic bread rolls [26]. Bread rolls with these characteristics exhibited a larger specific volume and a smaller hardness and were more acceptable to consumers than the rough, thick-walled, porous bread rolls. Furthermore, the improvement in gas retention and expansion was essential for achieving bread rolls with larger specific volumes, more pores, and superior viscoelastic properties. According to the related reports, the hydrophilicity of WOP was advantageous in increasing the water retention capacity of the dough, which could contribute to the formation of a more stable gluten network structure. As a result, the puffiness of the dough was improved, and the distribution of pores inside the bread rolls was more uniform [27].

The number of pores in the bread rolls containing 1.0% WOP was the largest, and their wall was the thinnest (*p* < 0.05), following a similar pattern to that of specific volume. The results indicated that the appropriate concentration of wheat peptides could improve the internal uniform tissue structure of bread rolls, form a more stable gluten network, and achieve homeostasis. However, there was no significant improvement in the bread roll quality when the addition of WOP exceeded 1.0% (i.e., 1.5%). Similar conclusions were also drawn regarding an excessive amount of protein hydrolysates (γ-[Glu] (1 ≤ *n* ≤ 5)-Gln, (GGP)) added to the bread rolls [28]. In our study, the excessive concentration of WOP (>1.0%) does not further improve the bread roll properties, which may be due to the damage to the gluten network structure, thus affecting the quality of the final product. To sum up, the incorporation of WOP resulted in a finer and more uniform internal structure of bread rolls, and it promoted gas production and retention in the dough. Specifically, the addition of WOP had the potential to improve bread roll quality, particularly at a supplemented proportion of 1.0%.

#### 3.1.3. Effects of WOP on the Hardness of Bread Roll Crumbs during the Storage Process

**Figure 2:**
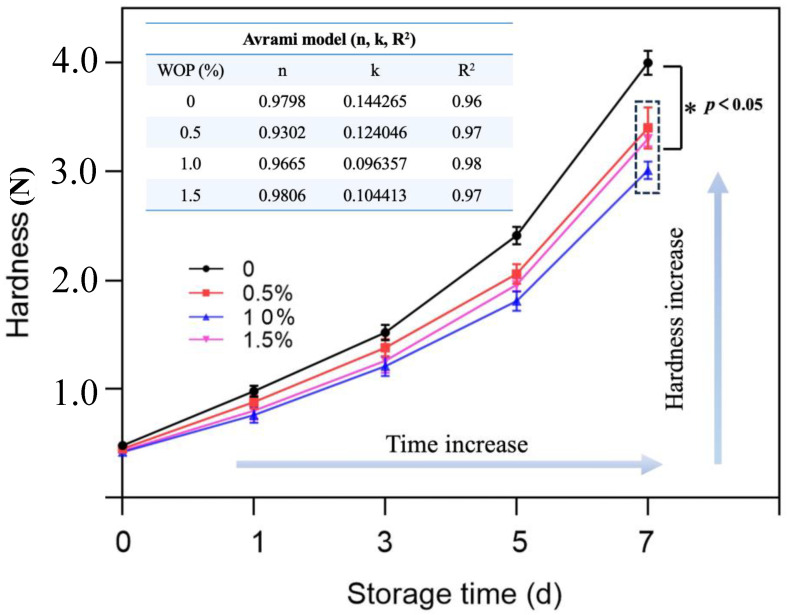
Effects of WOP (0–1.5%, *w*/*w*) on the hardness of bread roll crumbs stored at 4 °C for 7 days, and the Avrami model parameter was fitted based on the hardness.

Hardness is one of the most direct sensations that is experienced by people when they consume bread roll products, and the increase in hardness during the long-term storage process can cause a negative sensory experience. Therefore, it is necessary to explore the effect of WOP on the hardness of bread roll crumbs during their storage periods [5]. The hardness changes in bread roll crumbs with or without the addition of WOP after storage at 4 °C for 0, 1, 3, 5, and 7 days was shown in Figure 2. The results showed a significant increase in the hardness of bread roll crumbs at the maximum storage time (*p* < 0.05), which was one of the major physical phenomena indicating the bread staling [7,29]. It should be noted that after 3 days of storage, the hardness of bread roll crumbs increased dramatically from ~1.5 N (3 d) to ~4.0 N (7 d). Although the rearrangement of amylose molecules occurs mainly during the initial stages of bread roll storage, bread roll crumbs remain relatively moist and soft due to their higher moisture content. As the storage time increases, the water continues to evaporate and migrate to the crust and the starch molecules continue to retrograde, and this leads to a dramatic increase in the hardness of bread roll crumbs [30]. The addition of WOP reduces the hardness of bread roll crumbs during storage, which may also be related to the hydrophilicity of WOP, as it can hinder water absorption during starch gelatinization [31]. Moreover, the inclusion of WOP leads to a reduction in the hardness of bread roll crumbs during storage, which is closely correlated with the observed increase in specific volume. The incorporation of WOP contributes to the enhanced gas retention and volume expansion, resulting in a larger specific volume. This increased volume led to a reduction in hardness, as the bread possessed a softer and less dense texture.

Bread roll staling is a complex phenomenon that involves a variety of mechanisms, such as physical and chemical changes in the main bread roll components, such as protein, starch, and water, and their interaction during storage [17]. The WOP–starch aggregation may cause a denser gluten network and inhibit water change during the preparation of fermented dough [32]. According to a recent report, the incorporation of maize germ protein hydrolysate into bread roll inhibited the long-term retrogradation of starch by hindering the formation of amylopectin crystallites via a steric effect caused by the formation of ionic and hydrogen bonds, which could be the reason for the reduction in the hardness of bread roll crumbs during storage due to WOP [33]. In addition, the hydroxyl groups on the WOP molecule chain competitively hindered the aggregation and rearrangement of linear starch chains, as well as the formation of double helix structures in starch molecules [34].

Interestingly, the hardness of the bread roll crumbs did not decrease continuously with the increase in the WOP addition. Specifically, the hardness of bread roll crumbs was the lowest when the WOP addition reached 1.0%. After further increasing the concentration of WOP, the hardness slightly increased but was still lower than that of the control group (*p* < 0.05), indicating that WOP effectually delayed the staling behavior of bread roll during storage. Nevertheless, a high concentration of WOP (1.5%) potentially caused the water surrounding the amylopectin to bind, prompting a swift rearrangement of the crystalline regions [15]. Another possible explanation was that the excessive WOP could wrap around starch granules, reducing the air chambers in the gluten network and leading to a dense skeleton that supports the network, thus exhibiting a slightly higher hardness [35].

#### 3.1.4. Kinetics of the Retrogradation Process

The Avrami equation has been widely used to study the kinetic model of starch staling, which can be analyzed by the hardness value of bread rolls during storage [36]. The effect of WOP on the staling of bread roll was analyzed with the Avrami equation of hardness, and the parameters are shown in Figure 2. In this equation, ‘R^2^’ represented the fitting degree of the equation, the Avrami exponent ‘n’ was related to crystal nucleation and crystal size, and ‘k’ reflected the staling rate of the bread rolls.

As shown in Figure 2, the ‘R^2^’ of different gradients of WOP for bread rolls were all greater than 0.9, indicating that the Avrami model was fitted to kinetic hardness data [37]. The index *n* values were all less than 1.0, indicating that the nucleation mode of crystallization in bread rolls was mainly instantaneous nucleation, which was consistent with the previous report [34]. Moreover, exponent (n) largely defined the convergence towards the full crystallization/hardness potential energy [38]. The presence of WOP in the amylopectin crystals could lead to disturbed crystal growth, resulting in the lower *n* exponent obtained for this bread rolls [39]. The constant ‘k’ of the Avrami model was the rate constant of recrystallization, which reflected the staling rate of bread rolls. The ‘k’ of bread rolls with WOP was significantly reduced, which was attributed to WOP competing with starch for available water molecules and limiting the migration of water molecules. The migration of water molecules was restricted, which inhibited the recrystallization of starch [36]. Therefore, ‘k’ may also provide information about moisture diffusion, as low moisture diffusivity leads to low ‘k’ values [39]. Similar studies also showed that protein hydrolysates could reduce the staling rate of starch because the peptides and starch formed a tight aggregate that destroyed the continuous starch network [40]. Therefore, the internal structure of the bread rolls was denser as a result of the formation of the WOP–starch aggregates, which was beneficial in preventing the increase in hardness caused by moisture loss [12]. In summary, our findings suggested that WOP can retard the increase in the hardness of bread roll crumbs during the storage process.

### 3.2. Thermal Properties of Starch

**Figure 3:**
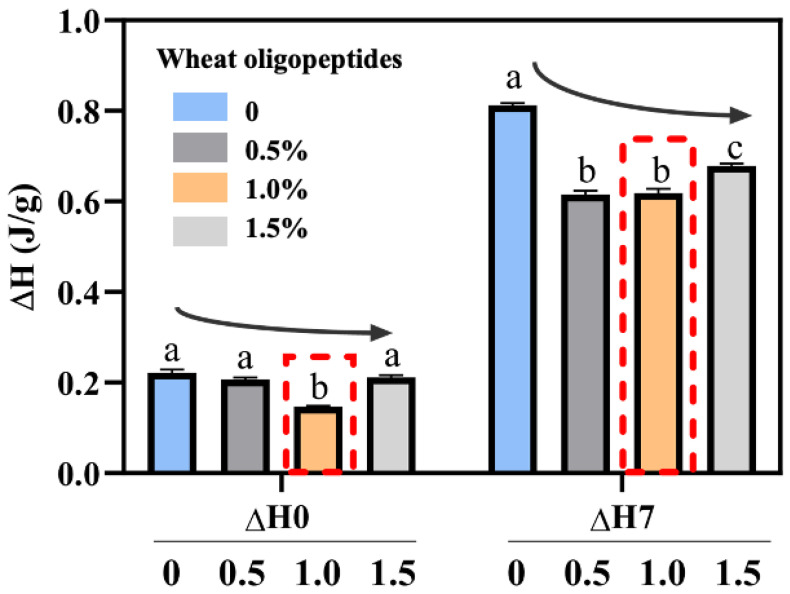
Effects of the addition of WOP (0–1.5%, *w*/*w*) on the thermal properties of bread rolls stored for 0 and 7 days. Note: ‘∆H’ represents the enthalpy change in gelatinization. Different lowercase letters as superscripts indicate significant differences in the same storage times (0 d or 7 d) with the different addition amounts (*p* < 0.05).

Starch retrogradation is the process of recrystallization of starch molecules through intermolecular hydrogen bonding during the storage of bread rolls [41]. The enthalpy changes (ΔH) were an indicator of starch molecules rearranging to form a new crystalline structure, which can reflect the degree of recrystallization of the starch molecule. To investigate the effect of WOP on starch crystallization, DSC was used to perform a thermodynamic analysis of bread rolls with different contents of WOP. As shown in Figure 3, compared with the control group, the incorporation of WOP reduced the ΔH of bread rolls. And with the addition of WOP (0–1.0%), the gelatinization enthalpy value decreased significantly (*p* < 0.05), which could be related to the hydration between WOP and starch [42]. Specifically, WOP could compete with starch for moisture because it is rich in hydrophilic amino acids, such as glutamic acid [43]. This can reduce the amount of water available for starch gelatinization, thereby reducing starch gelatinization. The addition of WOP promoted the formation of hydrogen bonds, and WOP surrounded the surface of starch molecules, effectively restraining starch swelling and reducing the gelatinization enthalpy [44].

After 7 days of storage at 4 °C, the enthalpy values of all bread rolls increased significantly (*p* < 0.05), indicating that starch recrystallized during storage, that is, bread roll staling occurred. Therefore, more energy was required to destroy the starch crystals, increasing the ΔH value. Compared with the control group, the ΔH value decreased with the increase in the WOP addition, and the staling enthalpy value of 1.0% WOP addition group was the lowest. The enthalpy of the bread rolls decreased from 0.812 J/g in the control group to 0.608 J/g after 7 days of storage. The results indicated that the addition of WOP reduced the amount of energy needed to break the interconnection of starch and water molecules. This might mean that WOP could reduce the availability of water molecules in the lattice formation and bonding process, as well as retard the migration of moisture molecules during amylopectin recrystallization, ultimately obstructing the starch crystallization [12]. The polyhydric structure of WOP likely contributes to the observed differences in ΔH. It is possible that the hydroxyl groups of WOP interact with the side chains of amylopectin and bind to the amorphous region of starch granules in varying degrees. As a result, these interactions may alter the coupling forces between the crystallites and the amorphous matrix within the starch, ultimately influencing the enthalpy changes [45]. Moreover, the addition of hydrophilic additives reduced the mobility and the heat transfer rates of water, which affected the staling behavior of starch.

### 3.3. Long-Range Ordered Structure Analysis Using XRD Method

The starch staling process was usually accompanied by the formation of the starch crystalline matrix. X-ray diffraction (XRD) can detect the structure and changes in starch crystals [46]; therefore, we used XRD to further study the addition of WOP on starch crystallization during bread roll storage.

**Figure 4:**
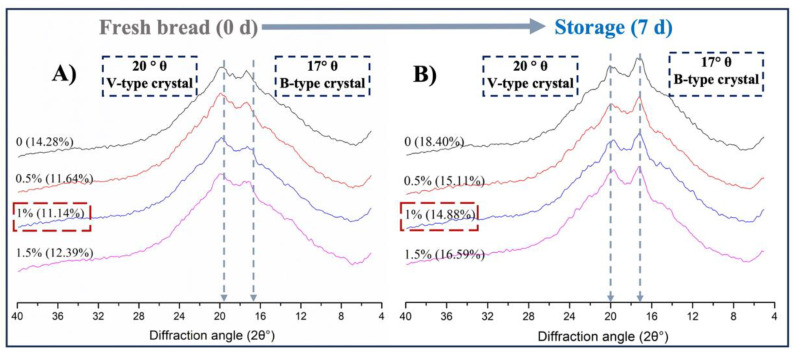
The X-ray diffraction (XRD) pattern of bread rolls with the addition of WOP (0–1.5%, *w*/*w*) at 4 °C for 0 d (**A**) and 7 d (**B**).

Figure 4 shows the XRD patterns of bread rolls with different added amounts of WOP after storage at 4 °C for 0 and 7 days. Two diffraction peaks were observed at 2θ near 17° and 20° for the fresh bread roll (0 day) samples. Among them, the peak at 20° indicated that the bread rolls formed V-shaped crystals after baking, representing the formation of the amylose–lipid complexes [47]. The formation of a peak at 17° indicated the formation of a B-type crystal. This was usually caused by the formation of double helixes between amylose and amylopectin during storage of gelatinized starch, which increased the crystallization proportion and the crystal integrity and reflected the degree of starch retrogradation [5].

The results showed that the positions of the diffraction peaks were not changed after the addition of WOP, indicating that no new starch crystal types were formed. However, the relative crystallinity of bread rolls decreased with the addition of WOP, especially the crystallinity of the bread rolls with 1.0% WOP decreased from 14.28% to 11.14%. The result indicates that WOP could delay the staling of bread rolls by inhibiting starch crystallization. After storage for 7 days, the intensity of the peaks located near 17° increased, due to the formation of an orderly structure of amylose and amylopectin [48]. The relative crystallinity of all bread roll samples increased when compared with that at 0 days, indicating the transformation of starch from an amorphous state to a polycrystalline state [49]. After 7 days of storage, the relative crystallinity of bread rolls with 1.0% WOP decreased to 14.88% compared with the 18.40% crystallinity of the non-WOP group. Similarly, other studies also showed that adding peptides, such as garlic peptides, could reduce the crystallinity of retrograded starch, which indicated that WOP had a similar effect on inhibiting the recrystallization of starch [42,50]. The above phenomenon showed that the degree of starch retrogradation in bread rolls increased during the storage process, and the addition of WOP slowed the retrogradation of starch, especially the recrystallization caused by amylopectin. In addition, according to a related study, some hydroxyl groups in the WOP interact with water molecules and starch molecules through hydrogen bonds, resulting in the interference of hydrophobic interaction between starch molecules, which is required for retrogradation [42,50].

Overall, the addition of WOP reduced the relative crystallinity of bread rolls, whether it was fresh bread rolls or bread rolls after 7 days of storage, indicating that WOP could inhibit the starch retrogradation of bread rolls during long-term storage, and the addition of 1.0% WOP showed the best effect. On the one hand, WOP could interact with starch to reduce the number of ordered structures of starch, thereby reducing the crystal regions in the mixed system [15]. On the other hand, this fact might be attributed to the crucial role water plays in starch recrystallization. The competition for water between WOP and starch results in a decrease in the water and amorphous regions available for binding to starch, thereby increasing the recrystallization area [51].

### 3.4. Short-Range Ordered Structure Analysis Using FTIR

**Figure 5:**
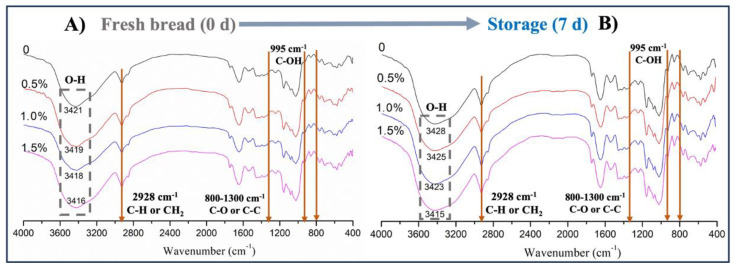
Effects of WOP (0–1.5%, *w*/*w*) on the FTIR spectra at 4 °C for 0 d (**A**) and 7 d (**B**).

The short-range order can reflect the variations in helicity, chain conformation, and double helix structure [52]. Fourier transform infrared spectroscopy (FTIR) can analyze the changes in the staling degree of starch during storage. The FTIR spectra of bread rolls with or without WOP addition at 4 °C storage for 0 d and 7 d are shown in Figure 5.

A broad and strong infrared absorption peak was observed around 3400 cm^−1^ for all samples, which was related to hydrogen bond formation. The peaks here corresponded to the stretching vibration of O-H in starch and the stretching vibration of N-H in protein [37]. Compared with the control group, the shape of the FTIR peak at 3400 cm^−1^ became wider and shifted to the lower wavenumber direction after the addition of WOP. This result indicated that WOP enhanced hydrogen bonding since it contained a large number of carboxyl groups, which formed hydrogen bonds by bonding with the hydroxyl groups of starch molecules, resulting in an enhanced absorption peak in this band [53]. In addition, the peak intensity at 2928 cm^−1^ increased with the addition of WOP, which may be caused by the C-H stretching vibration in amino acids or CH_2_ in starch, indicating the existence of glutamic acid in the hydrolysate [37]. The wavelength range of 800–1300 cm^−1^ was mainly related to C-O and C-C stretching vibrations, reflecting changes in polymer conformation and starch hydration, and the 995 cm^−1^ band was susceptible to changes in moisture content, mainly due to C-OH bending vibrations [54].

The bands at 1022 cm^−1^ and 1047 cm^−1^ reflected ordered regions and amorphous structures related to short-range ordered structures in starch [55]. The intensity ratio R_1047/1022_ was usually used as an index to evaluate the degree of order in starch. The intensity of the absorption peak at 995 cm^−1^ was related to the intramolecular hydrogen bonding of the hydroxyl group of C-6 [56]. The intensity ratio R_995/1022_ represented the order of double helixes, as shown in Table 1. After 7 days of storage, the values of R_1047/1022_ and R_995/1022_ showed an increasing trend, which was attributed to the rearrangement of amylopectin and the formation of an ordered structure [47]. The value of R_1047/1022_ was significantly reduced by the addition of WOP, especially at 1.0%. This result indicated that WOP increased the disordered structure of amylopectin and inhibited the rearrangement of amylopectin. The interaction between WOP and water molecules inhibited the aggregation between starch–water and starch–starch molecules. It promoted the destruction and dissociation of the double helix in amylopectin, which was reflected in the downward trend of R_1047/1022_ [57]. Moreover, the value of R_995/1022_ showed a similar trend, which reflected the decrease in the double helical molecular order of starch, indicating that WOP prevented the starch from recrystallizing. Additionally, the interaction of WOP and amylose was stronger than that of amylose and amylose, preventing the formation of hydrogen bonds between the leached amylose during the short-term retrogradation of wheat starch [58].

### 3.5. Moisture Content Determination

The moisture content in bread roll crumbs affected their texture, softness, and shelf life properties. The reduction and delay of moisture loss in bread roll crumbs during storage were generally beneficial in maintaining the softness of the crumbs, thereby ensuring a longer bread roll shelf life [7].

**Figure 6:**
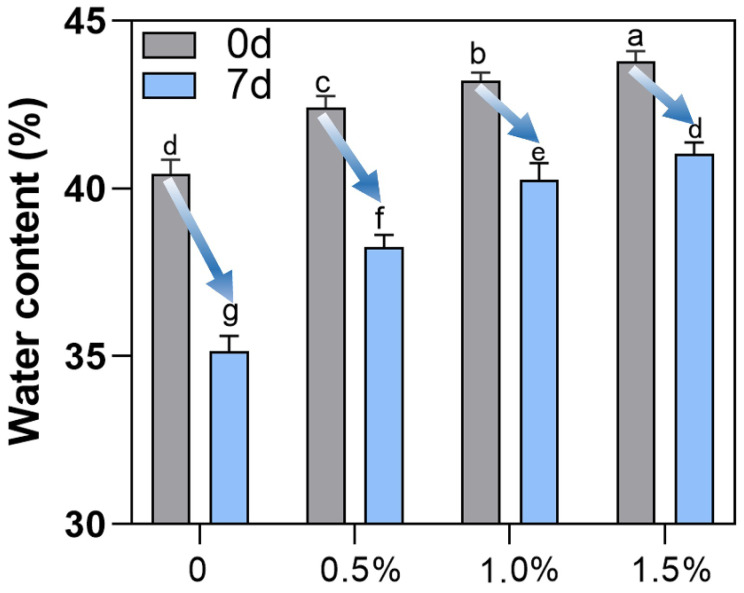
The effects of WOP (0–1.5%, *w*/*w*) on moisture content stored for 0 d and 7 d. Different lowercase letters as superscripts indicate significant differences in different storage times (0 d and 7 d) with the different addition amounts (*p* < 0.05). The same letter indicates no significant difference (*p* > 0.05).

The moisture content of the control group and the bread rolls with different contents of WOP during 0 and 7 days of storage were studied. As shown in Figure 6, the addition of WOP significantly increased the moisture content of the crumbs in fresh bread rolls (0 d), possibly due to the hydrophilic nature of WOP. After 7 days of storage at 4 °C, the moisture content of all samples decreased significantly (*p* < 0.05), but the moisture content of bread roll crumbs with WOP was always higher than that of the control bread rolls. Previous studies have shown that moisture content has a significant impact on bread roll quality [59]. Additives can decrease moisture loss, often by hindering moisture migrating from crumbs to the crust and subsequently evaporating into the surrounding environment.

A related study showed that the addition of maltitol to the bread rolls reduced the moisture loss during the storage period. Due to its hydrophilic structure, maltitol had a strong water-binding capacity during storage, retaining water in the bread rolls [19]. WOP is rich in hydrophilic groups and therefore readily binds to water by hydrogen bonding, limiting the mobility of water molecules. Studies have also shown that adding whey protein hydrolysates (WPH) can reduce the water mobility (T_2_ value) of rice starch during storage process and improve its water-holding capacity [34]. This may be due to the steric hindrance produced by WPH combined with starch chains, which hindered the binding of molecules between starch chains and the formation of hydrogen bonds, thereby reducing the exudation of water in gelatinized rice starch [34]. The migration of moisture involved the moisture transfer from bread roll crumbs to the crust and moisture migration from gluten to starch, thereby participating in the process of starch recrystallization.

### 3.6. SEM

In general, starch retrogradation and water loss lead to an increase in bread roll hardness, and the changes in bread roll texture (e.g., hardness) may also be associated with the changes in bread roll microstructure [5]. In order to better understand the role of WOP in delaying bread roll staling, the microstructure of bread rolls with different contents of WOP was observed using SEM.

**Figure 7:**
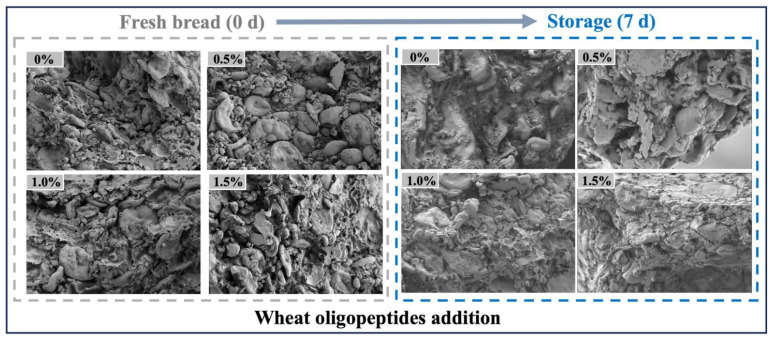
The effect of WOP (0–1.5%, *w*/*w*) on the microstructure of bread rolls stored for 0 d and 7 d.

The results in Figure 7 show the microstructure images of bread rolls with various concentrations of WOP after 0 and 7 days of storage at 4 °C. A continuous gluten network matrix could be observed in the microstructure of fresh control bread rolls, in which the spherical starch granules of different sizes were embedded in the gluten network [60]. The stability and strength of the network structure were crucial to the specific volume and internal texture of the final product. Compared with the control group, the roughness of the bread rolls was reduced when incorporated with WOP, the starch granules were more tightly embedded in the gluten network, the gluten structure was more complete, and there were fewer voids and pores [28]. This result implied that WOP was conducive to the formation of a more stable gluten network and a uniform internal structure of bread rolls, consistent with specific volume and C-Cell results. After 7 days of storage at 4 °C, the microstructure of the control bread rolls was looser, the gluten matrix became more discontinuous than at 0 day of storage, and the starch granules were exposed outside the gluten network structure. These changes revealed the occurrence of starch staling and the destruction of network structure. With the addition of WOP, the internal structure of the bread rolls was significantly improved, and the starch granules were still embedded in the gluten network structure quite uniformly. The network characteristics of the gluten matrix were more pronounced when the WOP content was 1.0%. The addition of WOP enhances the gluten network structure, effectively embedding in starch granules without creating voids, thus improving the microstructure of the bread rolls.

## 4. Conclusions

This study comprehensively elucidates the baking characteristics of bread rolls with WOP, the staling kinetics, thermal characteristics, and the mechanisms of anti-staling during storage. Our results showed that WOP significantly improved the baking quality of bread rolls and delayed adverse phenomena such as starch staling and moisture loss during the storage process, which demonstrated the potential application of WOP in bakery products. Based on our research findings, we recommend a WOP concentration of 1.0% to enhance bread roll quality. We are not yet to elucidate the moisture levels and moisture distribution throughout the bread roll storage process. The mechanism of moisture migration during the storage process will be the focus of our future exploration of bread roll staling. In conclusion, WOP can improve bread roll quality, extend its shelf life, and can be suitably used in the food industry. These results provided a theoretical basis for a further application of WOP in the food industry. These will expand the applications of WOP in the fields of anti-staling and quality improvement.

## Figures and Tables

**Table 1:** The effect of WOP (0–1.5%, *w*/*w*) on the FTIR peak ratio stored for 0 d and 7 d.

WOP (%)	Storage (d)	0	0.5	1.0	1.5
R_1047/1022_	0 d	0.891 ± 0.006 ^aA^	0.826 ± 0.008 ^aA^	0.726 ± 0.011 ^aB^	0.808 ± 0.005 ^aA^
	7 d	1.013 ± 0.008 ^bA^	0.956 ± 0.012 ^bB^	0.960 ± 0.008 ^bB^	0.971 ± 0.006 ^bB^
R_995/1022_	0 d	1.114 ± 0.005 ^aA^	1.030 ± 0.007 ^aA^	0.933 ± 0.009 ^aB^	1.044 ± 0.012 ^aA^
	7 d	1.323 ± 0.011 ^bA^	1.196 ± 0.005 ^bB^	0.954 ± 0.013 ^aC^	1.171 ± 0.009 ^aB^

Data are presented as the mean of replicate assays ± SD. Different lowercase letters as superscripts indicate significant differences in different storage times (0 d and 7 d) with the same addition amount (*p* < 0.05). Different uppercase letters indicate significant differences in the data under the same row. The same letter indicates no significant difference (*p* > 0.05).

## Data Availability

Data is contained within the article.
